# Host manipulation by bacterial type III and type IV secretion system effector proteases

**DOI:** 10.1111/cmi.13384

**Published:** 2021-08-30

**Authors:** Flávia Viana, Shruthi Sachidanandan Peringathara, Arshad Rizvi, Gunnar N. Schroeder

**Affiliations:** ^1^ Wellcome‐Wolfson Institute for Experimental Medicine, School of Medicine, Dentistry and Biomedical Sciences Queen's University Belfast Belfast, Northern Ireland UK

**Keywords:** bacterial pathogenesis, effectors, host‐pathogen interaction, infection, proteases, proteolysis, type III secretion system (T3SS), type IV secretion system (T4SS)

## Abstract

Proteases are powerful enzymes, which cleave peptide bonds, leading most of the time to irreversible fragmentation or degradation of their substrates. Therefore they control many critical cell fate decisions in eukaryotes. Bacterial pathogens exploit this power and deliver protease effectors through specialised secretion systems into host cells. Research over the past years revealed that the functions of protease effectors during infection are diverse, reflecting the lifestyles and adaptations to specific hosts; however, only a small number of peptidase families seem to have given rise to most of these protease virulence factors by the evolution of different substrate‐binding specificities, intracellular activation and subcellular targeting mechanisms. Here, we review our current knowledge about the enzymology and function of protease effectors, which Gram‐negative bacterial pathogens translocate via type III and IV secretion systems to irreversibly manipulate host processes. We highlight emerging concepts such as signalling by protease cleavage products and effector‐triggered immunity, which host cells employ to detect and defend themselves against a protease attack.

**Take Away:**

- Proteases irreversibly cleave proteins to control critical cell fate decisions.
- Gram‐negative bacteria use type III and IV secretion systems to inject effectors.
- Protease effectors are integral weapons for the manipulation of host processes.
- Effectors evolved from few peptidase families to target diverse substrates.
- Effector‐triggered immunity upon proteolytic attack emerges as host defence.

## Graphical abstract

Gram‐negative bacterial pathogens employ type III and IV secretion systems to translocate a multitude of diverse effector proteins into host cells to manipulate cellular processes to their benefit. This review summarises the enzymology and functions of effector proteases, which irreversibly cleave their targets, and discusses emerging concepts in protease detection and defence mechanisms of the hosts.

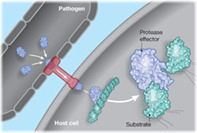

## 1 INTRODUCTION

Gram‐negative bacteria evolved virulence factors that enable them to compete with environmental predators, such as protozoa, or to exploit unicellular and higher organisms such as plants and animals as hosts. Protein secretion systems, particularly Type III and IV secretion systems (T3SS and T4SS), play a fundamental role in these interactions (Costa et al., 2015). T3SS and T4SS are highly optimised multi‐protein machineries that use distinct operation modes to translocate so‐called effector proteins from the bacteria directly into the host cell cytoplasm, enabling effective manipulation of host processes (Costa et al., 2021; Galan & Waksman, 2018). Diverse combinations of T3SSs and T4SSs are found in numerous bacteria, and they are key virulence factors for many clinically and economically important pathogens, including *Salmonella*, *Legionella* and *Pseudomonas* species (Abby et al., 2016; Buttner, 2012). Variable effector repertoires shape host and tissue tropism, virulence and pathology.

Effectors are structurally diverse, combining one or more activity domains with an essential, shared determinant, a translocation signal, encoded at the N‐terminus for T3SS or at the C‐terminus for T4SS effectors (Galan & Waksman, 2018). Activity domains can exert their effect on cellular processes by molecular mimicry of host proteins, for example, eukaryotic GTPase‐activating proteins (GAPs) to control the state of small GTPases (Elde & Malik, 2009) and/or have enzymatic activity. Many enzymatic activity domains manipulate proteins by introducing or removing post‐translational modifications (PTMs), e.g., phosphorylation or ubiquitination (Scott & Hartland, 2017; Tahir, Rashid, & Afzal, 2019). This overrides host control of protein activity, but as many PTMs are reversible, the duration of the manipulation depends on the availability and ability of cognate host proteins to counteract the effectors and restore normal protein states.

To irreversibly manipulate host processes, several pathogens deploy T3SS or T4SS protease effectors, which cleave a peptide bond in the polypeptide backbone of target proteins. An exception are deubiquitinase (DUB) effectors that cleave the isopeptide bonds between proteins and ubiquitin or ubiquitin‐like modifiers releasing the intact proteins (recently reviewed by (Kitao, Nagai, & Kubori, 2020; Kubori, Kitao, & Nagai, 2019). Here, we review the enzymology and functions of effector proteases of Gram‐negative bacterial pathogens, which irreversibly cleave the polypeptide backbone of target proteins (Table 1).

**TABLE 1:** Overview of the protease effectors discussed in this review

Name^a^	Uniprot ID^b^	Organism(s)^c^	SST	P T	M ID	Catalytic residues	PDB ID^d^	Substrate(s)/*interactor*	Function(s)/phenotypes
AnkH	Q5ZT65	*Legionella* spp.	IV	C	n.a.	H243 D258 C324	6MCA	*LARP7*	Modulation of transcription
AvrPphB	Q52430	*Pseudomonas syringae*	III	C	58	C98 H212 D227	1UKF	PBS1, RIPK, PBS1‐like kinases, BIK1	Inhibition of PTI
AvrRpt2	Q6LAD6	*Pseudomonas syringae*	III	C	70	C122 H208 D226		RIN4, NOIs, *Cyclophilin*	Inhibition of PTI, modulation of auxin signalling
AvrRpt2_EA_	A0A2U7NR52	*Erwinia amylovora*	III	C	70	C88 H173 D191	6HQZ	RIN4	Inhibition of PTI
EspL	B7UI20	EPEC, EHEC, *Citrobacter rodentium*	III	C	118	C47 H131 D153		RIPK1, RIPK3, TRIF, ZBP1	Inhibition of necroptosis
GogA	A0A0H3NQ02	*Salmonella enterica*	III	M	85	H182 E183 H185		RelA (p65), RelB	Inhibition of NF‐κB signalling
GtgA	A0A0F6AZI6	*Salmonella enterica*	III	M	85	H182 E183 H185	6GGO 6GGR	RelA (p65), RelB	Inhibition of NF‐κB signalling
GtgE	H9L447	*Salmonella enterica* (not Typhi)	III	C	102	C45 H151 D169	4MI7 5OED	Rab29, Rab32, Rab38	Inhibition of itaconate delivery to SCV
HopB1	G3XDC6	*Pseudomonas syringae*	III	T	8	T370 H413 D435 D436		Phospho‐BAK1 *FLS2*	Inhibition of PTI
HopN1	G3XDC5	*Pseudomonas syringae*	III	C	58	C172 H282 D299		PsbQ	Inhibition of stress response, ROS and callose deposition
HopX1	Q83YM6	*Pseudomonas syringae*	III	C	103	C179 H215 D233		JAZ proteins	Inhibition of SA‐induced defences
HopZ4	A0A3M4BU97	*Pseudomonas syringae*	III	C	55	C194 E152 H133		RPT6	Inhibition of SA‐induced defences
IpaJ	Q54150	*Shigella flexneri*	III	C	109^e^	C64 H206 D218		Myristoylated‐ARF/ARL GTPases	Inhibition of endocytic recycling and secretory pathway
LopT	Q7N4D9	*Photorhabdus luminescens*	III	C	58	C141 H259 D275		Rho GTPase	Inhibition of phagocytosis (likely)
Lpg1137	Q5ZWE8	*Legionella pneumophila*	IV	S	n.a.	S68		Synaxin 17	Inhibition of autophagy & apoptosis
NleC	Q8X834	EPEC, EHEC, *Citrobacter rodentium*	III	M	85	H183 E184 H187 D194	4Q3J 4O2I	RelA (p65), RelB, p105/50, p100/p52, c‐Rel, p300	Inhibition of NF‐κB signalling
NleD	B7UNX6	EPEC, EHEC, *Citrobacter rodentium*	III	M	91	H142 E143 H146		JNK, p38	Inhibition of NF‐κB signalling and apoptosis
NopT_SF_	P55730	*Sinorhizobium fredii*	III	C	58	C93 H205 D220			Regulation of symbiosis, nodule formation
NopT_MA_	n.a.^f^	*Mesorhizobium amphore*	III	C	58	C97 H210 D225		*ATP‐CSACP2 HIRP*	Inhibition of plant immune signalling
OspD3	A0A4P7TTX1	*Shigella flexneri*	III	C	118	C64 H148 D171		RIPK1, RIPK3	Inhibition of necroptosis
PipA	A0A0H3NFC2	*Salmonella enterica*	III	M	85	H180 E181 H184		RelA (p65), RelB	Inhibition of NF‐κB signalling
RavK	Q5ZWW5	*Legionella pneumophila*	IV	M	n.a.	H95 E96 H99		Actin	Disruption of Actin cytoskeleton
RavJ	Q5ZWY9	*Legionella pneumophila*	IV	C	n.a.	C101 H138 D170	4XA9	*LegL1/Septins*	Cytotoxic for yeast, affects Actin cytoskeleton
RavZ	Q5ZUV9	*Legionella pneumophila*	IV	C	n.a.	C258 H176 D197	5MS2	Myristoylated LC3	Inhibition of autophagy
RipE1	Q8XU25	*Ralstonia solanacearum*	III	C	103	C172 H203 D222		JAZ proteins	Inhibition of SA‐induced defences
RipT	Q8XUH6	*Ralstonia solanacearum*	III	C	58	C123 H245 D262			
SpvD	P0A2N2	*Salmonella enterica*	III	C	117	C73 H162 D182	5LQ6	*Xpo2*	Inhibition of NF‐κB signalling
VPA1380	Q87GD7	*Vibrio Para‐haemolyticus*	III	C	n.a.	C195 H154			Cytotoxic in yeast
XopD	Q3BYJ5	*Xanthomonas campestris*	III	C	48	H249 D314 C355	5JP1 5JP3	Ubiquitin/SUMO‐SIERF4	Inhibition of PTI/ stress response signalling
XopJ	Q3BTM6	*Xanthomonas campestris*	III	C	55	C235		RPT6	Inhibition of SA‐induced defences
YopT	O68703	*Yersinia spp*.	III	C	58	C139 H258 D274		Prenylated RhoA, RhoG, Cdc42, Rac1	Disruption of cytoskeletal dynamics, inhibition of cell migration and phagocytosis

Abbreviations: M ID, MEROPS ID; n.a., not assigned; PT, protease type; SST, secretion system type.

^a^ Most common name, which was used in this review, synonyms might exist.

^b^ ID of representative protein in Uniprot database https://www.uniprot.org/.

^c^ Organism/Group of organisms, in which the prototype protease was described.

^d^ PDB database ID for representative 3D structure with and without substrate if available https://www.rcsb.org.

^e^ Preliminary MEROPS classification under review https://www.ebi.ac.uk/merops.

^f^ NCBI Refseq non‐redundant protein accession number: WP_040582237.1.

## 2 ENZYMOLOGY AND CLASSIFICATION OF PROTEASES

**FIGURE 1:**
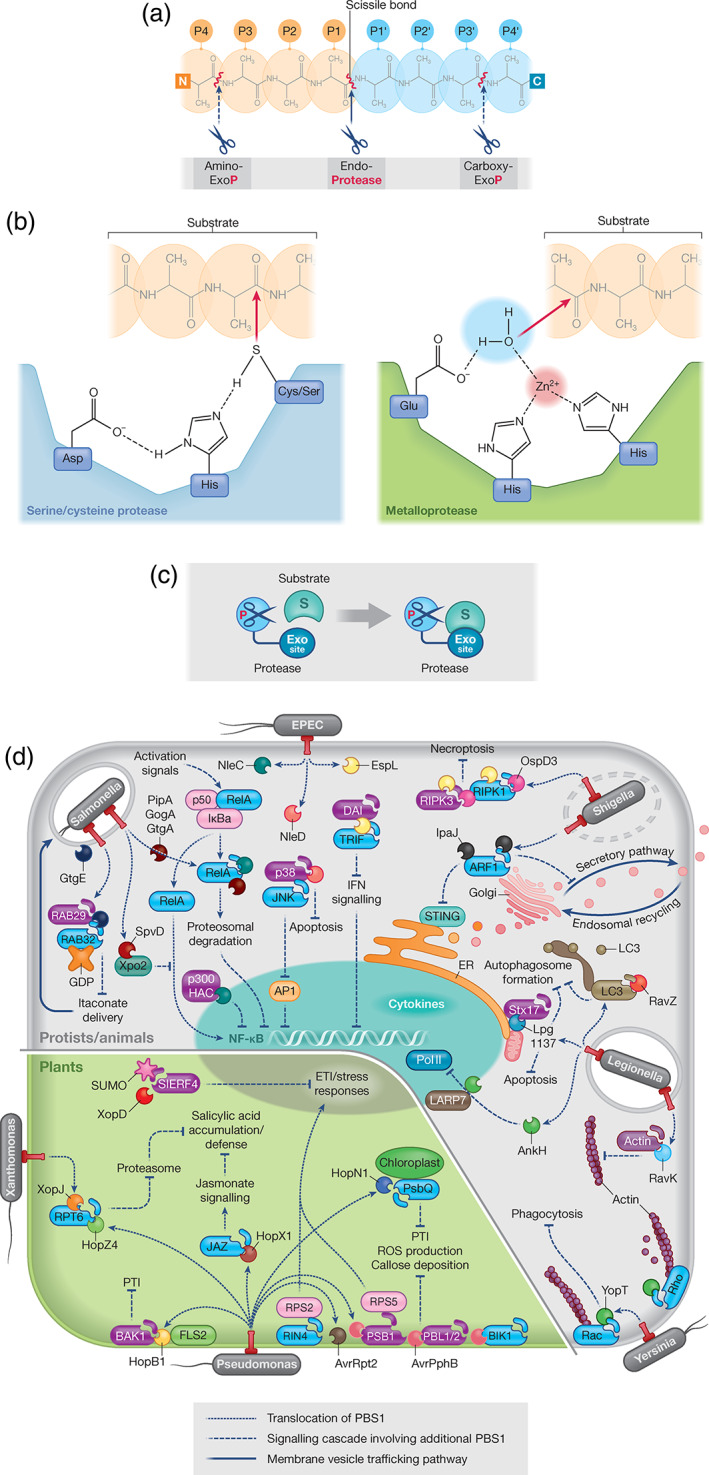
(a) Scheme illustrating the terminology used to describe protease cleavage specificity. Endopeptidases catalyse the hydrolysis of a peptide bond in the central region, exopeptidases close to the N‐ or C‐terminus of a substrate and are also termed aminopeptidases or carboxypeptidases, respectively. To describe the cleavage site sequence specificity, the residues N‐terminal to the cleaved bond are designated P1, P2, etc. and the ones C‐terminal P1′, P2′, etc., with numbers increasing with distance to the scissile bond. (b) Scheme of the active sites of serine/ cysteine‐ and metalloproteases. Serine and cysteine proteases rely on a catalytic triad, consisting of a serine or cysteine, a histidine and a third residue, aspartate, asparagine or glutamate. The residues are found in a spatially conserved constellation, facilitating deprotonation of the hydroxyl (serine) or thiol (cysteine) groups via the histidine, activating them for a nucleophilic attack on the carbonyl carbon of the peptide bond. The resulting covalent enzyme‐substrate intermediate is hydrolysed by a water molecule releasing two peptide fragments and the protease. Metalloproteases rely on one or two divalent metal ion co‐factors, for example, Zn^2+^, which polarise a water molecule, priming it for a nucleophilic attack, and the substrate carbonyl group, making its carbon more electrophilic and susceptible to the water molecule. The attack leads to a non‐covalent intermediate which resolves to release the cleavage products. (c) Scheme depicting the role of protease exosites in substrate recruitment. (d) Illustration highlighting the different activities of the discussed protease effectors in host cells

Proteases are believed to have evolved in primaeval organisms as destructive enzymes mediating protein catabolism and production of amino acids (Neurath, 1984), but their vital roles in cell signalling and physiology are now recognised (Lopez‐Otin & Bond, 2008). They are classified based on sequence and structural homology, cleavage specificity and catalytic mechanism (Rawlings, 2013), information which is captured in the MEROPS database (https://www.ebi.ac.uk/merops/, Rawlings et al., 2018]). Key terminology to describe the cleavage specificity is illustrated in Figure 1a. Proteolysis requires the catalytic activation of the target peptide bond and/or an incoming water molecule to enable hydrolysis. Proteases have evolved different substrate binding pockets and catalytic mechanisms to facilitate this. Six major classes are distinguished: aspartic, glutamic, threonine, metallo‐, serine and cysteine proteases (Rao, Tanksale, Ghatge, & Deshpande, 1998; Rawlings, 2013). All identified effector proteases, but one mechanistically not fully characterised threonine protease belong to the last three classes. Within these classes, a few peptidase families seem to have given rise to the vast majority of effectors. The expansion of these families might have been promoted by horizontal gene transfer and subsequent functional diversification, an important driver for the evolution of virulence (Diard & Hardt, 2017). Adaptation of existing effectors for new purposes seems favourable compared to the evolution of new effectors from non‐effector genes, which would require, for example, the acquisition of secretion signals and integration into the regulatory circuits controlling virulence. In addition, unknown intrinsic features might make these peptidases in particular suitable for their use as effectors. However, as we only start to reveal the functions of effectors from environmental pathogens such as *Legionella pneumophila*, which have much larger effector arsenals than host‐restricted pathogens and a high propensity to convert host cell genes to effectors, additional effector peptidase families are likely to be discovered.

Figure 1b depicts common active sites of serine and cysteine proteases, which rely on a catalytic triad consisting of a serine or cysteine, a histidine and a third residue, often aspartate (Rao et al., 1998; Rawlings, 2013), and metalloproteases, which depend on one or two divalent metal ion co‐factors for catalysis (Cerda‐Costa & Gomis‐Ruth, 2014). Variations of this catalytic core occur; for example, some cysteine proteases only require cysteine and histidine residues. Mutation of the catalytic triad or metal‐coordinating residues renders these proteases usually inactive. The substrate specificity is determined by the active site binding pocket, but often also by exosites, which recruit the substrates and can also control the subcellular localisation of proteases (Figure 1c). Production of inactive pro‐enzymes, which only get activated in the host, and secretion of finite amounts of effector during a limited time window are additional mechanisms employed by pathogens to exert control of protease activity. Despite their destructive potential, most effector proteases perform selective manipulations of the host (Figure 1d).

## 3 EFFECTOR PROTEASES OF BACTERIAL ENTEROPATHOGENS

Enteropathogenic and enterohemorrhagic *Escherichia coli* (EPEC & EHEC), *Salmonella*, *Shigella*, *Yersinia* and *Vibrio* spp. cause a wide spectrum of diarrheal diseases and are a global health problem (Collaborators, 2018). They all rely on T3SSs as key virulence factors to deliver distinct repertoires of effectors to shape their pathogenesis. However, due to shared evolutionary origins and horizontal gene transfer, homologue effectors are often found in several enteropathogens (Pinaud, Sansonetti, & Phalipon, 2018).

### 3.1 Disruption of NFκB and MAPK‐driven immune signalling by protease effectors

EPEC, EHEC, *Citrobacter rodentium* and *Salmonella* spp. are closely related pathogens but colonise different niches in human and animal hosts. EPEC, EHEC and *C*. *rodentium*, a mouse‐adapted relative and surrogate model for EPEC and EHEC (Collins et al., 2014), are extracellular pathogens, which use Locus of enterocyte effacement T3SS‐secreted effectors to attach to intestinal epithelial cells inducing characteristic attaching and effacing (A/E) lesions (Wong et al., 2011). *Salmonella enterica* subspecies are facultative intracellular gastrointestinal pathogens. Two T3SSs encoded by the *Salmonella* pathogenicity islands (SPI)‐1 and SPI‐2 mediate invasion of host cells (SPI‐1) and establishment of the *Salmonella*‐containing vacuole (SCV) (SPI‐2), in which the bacteria replicate (Ibarra & Steele‐Mortimer, 2009). Despite the different infection strategies, these pathogens deploy effectors from two related zinc‐metalloprotease families, the NleC and NleD families, which share essential HExxH metal‐binding motifs but limited overall sequence similarity with other metalloproteases (Figure 1b) (Baruch et al., 2011; Marches et al., 2005; Pearson, Riedmaier, Marches, Frankel, & Hartland, 2011; Yen et al., 2010).

NleD from EPEC and EHEC (Marches et al., 2005) and its homologues in *C*. *rodentium*, *S*. *enterica* serovar Arizonae and in the endosymbiont *Hamiltonella defensa* target the Mitogen‐activated protein kinases (MAPKs) JNK and p38, but not ERK (Baruch et al., 2011; Gur‐Arie, Eitan‐Wexler, Weinberger, Rosenshine, & Livnah, 2020). Activities of homologues in the plant and insect pathogens *Pseudomonas syringae* (HopPtoH), *Xanthomonas campestris* (XCC3258), *Ralstonia solanacearum* (RSc3290 [RipAX1], RS03907) remain unknown (Marches et al., 2005). Cleavage occurs in the unstructured, conformationally‐flexible activation loops of the two MAPKs, in (JNK) or in proximity (p38) of a highly conserved TXY motif (Creuzburg et al., 2017; Gur‐Arie et al., 2020). Higher conformational rigidity protects the activation loop of ERK from cleavage, providing a remarkable example of entropy‐driven protease selectivity (Gur‐Arie et al., 2020). JNK and p38 orchestrate a wide variety of host cell responses downstream of cell surface receptors for cytokines and pathogen‐associated molecular patterns (PAMPs), such as Toll‐like receptors (TLRs) and tumour necrosis factor receptors (TNFR). Experiments exploiting a mutant of NleD, which fails to cleave p38 but not JNK, show that cleavage of p38 underlies the suppression of IL‐6 production upon EPEC infection (Creuzburg et al., 2017). JNK cleavage is associated with reduced pro‐apoptotic signalling (Baruch et al., 2011).

NleC is the prototype of the second important zinc metalloprotease effector family conserved in many A/E pathogens. Each *S*. *enterica* isolate encodes at least one of three related proteases, PipA, GtgA and GogA (Sun, Kamanova, Lara‐Tejero, & Galan, 2016), which are encoded in SPI‐5 (PipA) (Wood et al., 1998) or within lysogenic phages (GtgA, GogA) (Figueroa‐Bossi, Uzzau, Maloriol, & Bossi, 2001). All four effectors target members of the NF‐κB family of transcription factors, with NleC having the widest substrate spectrum cleaving NF‐κB RelA (p65), RelB, p105/50, p100/p52 and c‐Rel (Baruch et al., 2011; Pearson et al., 2011; Yen et al., 2010). In addition, NleC cleaves the acetyltransferase and transcriptional coactivator p300 in its kinase‐inducible domain (KID) interacting domain (KIX) (Shames et al., 2011). NleC suppresses proinflammatory signalling, for example, IL‐8 release by EPEC‐infected cells, and NF‐κB signalling, immune cell infiltration and tissue damage in *C*. *rodentium*‐infected mice (Baruch et al., 2011; Hodgson et al., 2015; Marches et al., 2005; Pearson et al., 2011; Ruano‐Gallego et al., 2021; Sham et al., 2011; Shames et al., 2011; Yen et al., 2010). However, neither NleC nor NleD is required for colonisation of mice by *C*. *rodentium* (Ruano‐Gallego et al., 2021; Sham et al., 2011) or for the persistence of EHEC O157:H7 in lambs or calves (Marches et al., 2005). The N‐terminal fragment of cleaved p65 amplifies the inhibition of NF‐κB signalling by disrupting the interaction of p65 DNA‐binding complexes with the ribosomal protein S3 (RPS3), which otherwise enhances the transcription of selected proinflammatory genes (Hodgson et al., 2015).

PipA, GtgA and GogA only cleave RelA (p65), RelB and c‐Rel (Sun et al., 2016). Structural and mutational analysis of GtgA and the related proteases revealed that these effectors, as NleC (W. Li et al., 2014; Turco & Sousa, 2014), have a negatively charged active site cleft, which mimics DNA and facilitates binding of the Rel homology region of NF‐kB subunits, but different P1′ residue preferences that alter substrate selectivity (Jennings, Esposito, Rittinger, & Thurston, 2018). While NleC cleaves between Cys‐38 and Glu‐39 of p65, a conserved site in all NF‐κB family substrates (Hodgson et al., 2015; Yen et al., 2010), PipA, GtgA, and GogA cleave between Gly‐40 and Arg‐41 of RelA (p65), failing to process NF‐κB1 (p100) and NF‐κB2 (p105), which contain a proline instead of Arg‐41 (Jennings et al., 2018; Sun et al., 2016). Biochemical data suggest that PipA, GtgA and GogA could be functionally redundant. In a murine infection model, a *S*. *Typhimurium pipA*, *gogA* and *gtgA* triple mutant was not attenuated in colonisation, albeit causing more severe inflammation (Sun et al., 2016). However, another study proposes that different temporal regulation of expression and translocation results in unique roles, for example, for PipA, in promoting systemic infection (Takemura, Haneda, Idei, Miki, & Okada, 2021). Further clarification of the individual roles of these proteases in *Salmonella* pathogenesis is needed. Uncharacterised homologues of NleC exist in diverse bacteria, for example, *Arsenophonus nasoniae* and *Yersinia aldovae* (Pearson et al., 2011) and, notably, the T2SS secreted AB‐toxin AIP56 of *Photobacterium damselae*, a pathogen of marine animals, also contains a NleC‐like domain and cleaves p65, showing that NleC‐like proteases are widespread and incorporated into diverse virulence factors (Silva et al., 2013).

*Salmonella enterica* also uses the SPI‐2 effector SpvD to inhibit NF‐κB‐driven pro‐inflammatory signalling, contributing to full virulence in a murine infection model (Grabe et al., 2016; Rolhion et al., 2016). SpvD is structurally related to CA clan papain‐like proteases (Barrett & Rawlings, 1996), the largest family of cysteine proteases, which have evolved to target a huge diversity of substrates. Its activity in vitro and vivo depends on its catalytic triad (Grabe et al., 2016). In vitro, SpvD variants from different *Salmonella* isolates hydrolyse an RLRGG peptide‐based small‐molecule substrate indicative of DUB‐like activity, but only one variant shows activity towards a full‐length ubiquitin‐probe (Grabe et al., 2016). SpvD targets but does not cleave cytoplasmic Exportin‐2 (Xpo2), derailing nuclear transport and shuttling of p65 into the nucleus (Grabe et al., 2016; Rolhion et al., 2016). Hence, the exact mechanism of action of SpvD during infection remains elusive.

### 3.2 Inhibition of necroptosis by EspL‐type cysteine proteases

The EPEC effector EspL also belongs to the papain‐like CA clan proteases (Pearson et al., 2017). EspL cleaves a conserved QxGxx↓N (P5‐P4‐P3‐P2‐P1‐P1′) sequence motif within Receptor interacting protein (RIP) homotypic interaction motif (RHIM) domains. In vitro, the RHIM containing proteins Receptor‐interacting serine/threonine‐protein kinase 1 (RIPK1), Receptor‐interacting serine/threonine‐protein kinase 3 (RIPK3), TIR‐domain‐containing adapter‐inducing interferon‐β (TRIF) and Z‐DNA‐binding protein 1 (ZBP1/DAI) are cleaved, but not RIPK2, which lacks a RHIM domain (Pearson et al., 2017). RIPKs play important roles in the regulation of inflammation, autophagy and necroptosis, a caspase‐independent form of programmed cell death (Eng, Wemyss, & Pearson, 2021). During EPEC infection, cleavage of RIPK1 and RIPK3 occurs, most likely before their oligomerisation and necrosome formation. Combined with TRIF cleavage, this prevents necroptosis and inflammatory signalling in response to TNFα and the TLR ligands polyinosinic: polycytidylic acid and lipopolysaccharide (Pearson et al., 2017). *C*. *rodentium* lacking its EspL homologue shows reduced colonisation of mice, confirming its contribution to full virulence (Pearson et al., 2017). EspL‐like proteins are encoded in a range of bacteria, including *Yersinia* and *Shigella* spp. (Pearson et al., 2017); however, only *Shigella* homologues have been characterised.

*Shigella* spp. are human‐adapted facultative intracellular pathogens, which use the Mxi/Spa T3SS to invade the cytoplasm and subvert macrophages and intestinal epithelial cells, causing shigellosis, which typically manifests as severe diarrhoea (Schroeder & Hilbi, 2008). *Shigella* spp. encode three OspD proteins related to EspL and, one of them, OspD3, also cleaves RIPK1 and RIPK3, blocking necroptosis (Ashida, Sasakawa, & Suzuki, 2020). OspD2 reduces *Shigella*‐induced cell death, but this is independent of its putative catalytic motif and rather due to intrabacterial activity limiting the secretion of other T3SS effectors, particularly VirA (Mou, Souter, Du, Reeves, & Lesser, 2018). OspD1 represses transcription of a set of effectors until secreted by the T3SS (Parsot et al., 2005). Functions within host cells for OspD1 and OspD2 have not been established yet.

## 4 DISRUPTION OF SMALL GTPASE FUNCTIONS BY PROTEASE EFFECTORS

The multitudinous Ras‐family of small GTPases controls numerous essential cellular processes, including cytoskeletal dynamics and membrane trafficking (Wennerberg, Rossman, & Der, 2005) and members are therefore prime targets for pathogens.

The papain‐like CA clan protease effector GtgE is encoded on the Gifsy‐2 bacteriophage, which is distributed among *S*. *enterica* Typhimurium and other wide‐host range *Salmonella* serovars, but not in the human restricted *S*. Typhi and *S*. Paratyphi (Ho et al., 2002; Kohler, Spano, Galan, & Stebbins, 2014; Spano, Liu, & Galan, 2011; Xu, Kozlov, Wong, Gehring, & Cygler, 2016). This effector cleaves the GTPases Rab29, Rab32 and Rab38, but not the related Rab40B, Rab23, Rab5 and Rab7, inactivating them and preventing their accumulation on the SCV (Kohler et al., 2014; Spano & Galan, 2012; Spano, Liu, & Galan, 2011). GtgE cleaves specifically the GDP‐bound Rab32, which is facilitated by the effector SopD2, a Rab32 GAP (Spano, Gao, Hannemann, Lara‐Tejero, & Galan, 2016; Wachtel et al., 2018). Cleavage occurs after glycine 41 (Rab29), 59 (Rab32) and 43 (Rab38) in the switch I region, which during the GTPase cycle undergoes a conformational change, controlling interactions of the RabGTPases with host partners. Cleavage results in a stable product, which still interacts with Rab GDP dissociation inhibitor, allowing membrane extraction, but not with all cognate host binding partners (Savitskiy et al., 2021; Wachtel et al., 2018). Inactivation of Rab32 by GtgE impedes the delivery of the antimicrobial metabolite itaconate from the mitochondria to the SCV, promoting bacterial survival in murine macrophages and mice (Chen et al., 2020).

Remarkably, gain and loss of function experiments demonstrate that due to the lack of GtgE, *S*. Typhi cannot interfere with the Rab32‐mediated defence mechanism in murine macrophage and thus cannot establish an infection (Spano & Galan, 2012); however, *S*. Typhi blocks the pathway in human macrophages in a SPI‐1‐dependent manner, suggesting that it uses other unidentified effectors (Baldassarre et al., 2021). This illustrates how effectors can shape the host range of pathogens.

While GtgE cleaves the GTPases in the switch I region, other protease effectors interfere with the PTM of small GTPases with lipid anchors, which are critical for membrane association and spatiotemporal control of their activity.

Invasion plasmid antigen J (IpaJ), a T3SS cysteine protease effector of *S*. *flexneri*, cleaves many proteins in vitro (Burnaevskiy et al., 2013), but during infection displays high specificity for myristoylated, Golgi‐localised GTPases of the ARF/ARL family (ARF‐1, −3, −4, −5, Arl‐1, −4c) (Burnaevskiy, Peng, Reddick, Hang, & Alto, 2015). IpaJ recognises specifically the GTP‐bound, myristoylated form of ARF1 and cleaves the peptide bond after the N‐terminal myristoylated Gly residue, removing the lipid anchor. This contributes to the disruption of the Golgi apparatus in infected cells, derailing the secretory pathway, endocytic recycling and cytokine secretion, as well as the translocation of STING, an intracellular innate immune sensor for microbial cyclic dinucleotides, from the endoplasmic reticulum (ER) to the ER–Golgi intermediate compartment (ERGIC), inhibiting IFN‐β induction in response to *Shigella* infection (Burnaevskiy et al., 2015; Dobbs et al., 2015).

*Yersinia* outer protein T (YopT) is a T3SS cysteine protease effector produced by many pathogenic *Yersinia* species, for example, *Y*. *pestis*, *Y*. *pseudotuberculosis* and *Y*. *enterocolitica* (Iriarte & Cornelis, 1998; Palace, Proulx, Szabady, & Goguen, 2018). YopT cleaves directly before the prenylated C‐terminal cysteine of RhoGTPases RhoA, Rac, Cdc42 and RhoG independently of the activation state, releasing them from the membrane (Mohammadi & Isberg, 2009; Shao, Vacratsis, et al., 2003; Shao, Merritt, Bao, Innes, & Dixon, 2002). Prenylation and a polybasic region in the Rho GTPases are key for recognition and processing (Fueller & Schmidt, 2008; Shao, Vacratsis, et al., 2003), and small differences in these elements lead to different turnover rates, making RhoA the preferred substrate (Fueller & Schmidt, 2008); however, during infection substrate selection might be influenced by cell‐type‐specific expression levels of the GTPases and other effectors, in particular YopE, which is a Rho GAP (Von Pawel‐Rammingen et al., 2000). RhoA, Rac and Cdc42 have important functions in coupling cytoskeletal and membrane dynamics and microinjection of YopT causes cell rounding and death (Iriarte & Cornelis, 1998; Sorg, Burns, Goehring, Aktories, & Schmidt, 2001). In cellular infection models, YopT contributes to the inhibition of uptake into non‐phagocytic and phagocytic cells and interferes with podosomal adhesion structures required for macrophage migration (Adkins et al., 2007; Aepfelbacher et al., 2003; Grosdent, Maridonneau‐Parini, Sory, & Cornelis, 2002). While YopT obstructs functions of the GTPases at the membrane, it generates distinct cytosolic and nuclear pools of the delipidated but active proteins, for example, Rac1, which could have biological functions (Wong & Isberg, 2005). In macrophages, enzymatically active YopT augments TNFα production in response to T3SS activity (Auerbuch, Golenbock, & Isberg, 2009) and stimulates a pyrin‐dependent innate immune surveillance pathway, which senses the inactivation of RhoA and drives inflammasome activation leading to proinflammatory cytokine release and pyroptosis (Medici, Rashid, & Bliska, 2019). This effector‐triggered immunity (ETI) (Lopes Fischer, Naseer, Shin, & Brodsky, 2020) might explain why YopT has only a minor role (Palace et al., 2018; Viboud, Mejia, & Bliska, 2006) or even decreases virulence (Trulzsch, Sporleder, Igwe, Russmann, & Heesemann, 2004) in murine infection models for different *Yersinia* species. ETI is a well‐established mechanism in plants and emerging for animals, sensing not only protease cleavage or other modifications of effector targets but also disruption of, for example, ER homeostasis or depletion of nutrients (Lopes Fischer et al., 2020). It enables the host to mount a pathogen‐specific response beyond the basal immune response elicited by PAMPs such as LPS, which are also found in non‐pathogenic bacteria. ETI explains why the deletion of some effectors can result in enhanced virulence. ETI sensors for many effectors may exist but might have gone unnoticed because, in the arms race between host and pathogen, the bacteria in turn, acquired effectors to block ETI (Thordal‐Christensen, 2020). *Yersinia* for example counteracts pyrin signalling using the effector YopM (Medici et al., 2019). These intricate interdependencies of effectors can lead to stable effector networks, which cannot be randomly expanded or reduced (Ruano‐Gallego et al., 2021).

YopT is the prototype of the peptidase C58 family (Shao et al., 2002). Homologous T3SS effectors are found in plants and other pathogens but, apart from the effector LopT from the entomopathogen *Photorhabdus luminescens* (Brugirard‐Ricaud et al., 2005) seem to have evolved different substrate profiles (see below). Peptidase C58 domains exist in other proteins, for example, *L*. *pneumophila* T4SS effectors (Schroeder et al., 2010) and large toxins such as PaTox and MARTX (Bogdanovic et al., 2019), but their physiological activities still need to be defined. Interestingly, MARTX from *Vibrio cholerae* contains an additional cysteine protease domain (CPD), which is required for auto‐processing upon binding of the host factor inositol hexakisphosphate (IP6) (Egerer & Satchell, 2010). The T3SS effector VPA1380 of *Vibrio parahaemolyticus*, a marine and foodborne human pathogen, which causes gastroenteritis (Broberg, Calder, & Orth, 2011), shares similarity with CPDs and inhibits the growth of yeast in a catalytic cysteine and IP6 dependent manner, but actual proteolytic activity of VPA1380 still needs to be validated (Calder et al., 2014). The presence of these related protease domains in effectors and secreted toxins suggests that they are common building blocks of new virulence factors.

## 5 T4SS EFFECTOR PROTEASES IN HOST SUBVERSION BY *LEGIONELLA PNEUMOPHILA*

*Legionella pneumophila*, the causative agent of the potentially fatal pneumonia Legionnaires' disease, translocates more than 330 effectors through its Defect in organelle trafficking/Intracellular multiplication (Dot/Icm) T4SS to establish a replication‐permissive niche, the *Legionella* containing vacuole (LCV), in macrophages and protozoa (So, Mattheis, Tate, Frankel, & Schroeder, 2015). Key for the intracellular lifestyle is the evasion of phagolysosomal degradation and innate defence mechanisms, such as autophagy, which can trap intracellular bacteria in membrane‐bound compartments that mature into the degradative auto‐phagolysosomes (Huang & Brumell, 2014). *L. pneumophila* uses effector proteases to interfere with several host processes.

The serine protease effector Lpg1137 cleaves syntaxin 17 (Stx17) (Arasaki et al., 2017), which is required to initiate autophagosomal membrane nucleation by recruitment of a phosphatidylinositol 3‐phosphate (PI3P) kinase (PI3K)‐ATG14L‐Beclin1 complex to mitochondria‐ER contact sites (Hamasaki et al., 2013) and later in the maturation process facilitates the fusion of autophagosomes with lysosomes (Viret & Faure, 2019). Stx17 also regulates the GTPase Drp1, which controls mitochondrial dynamics and Bax‐dependent apoptosis. Ectopically‐expressed Lpg1137 disrupts autophagosome nucleation and blocks staurosporine‐induced apoptosis, suggesting that the effector might serve multiple purposes during infection (Arasaki et al., 2017).

After membrane nucleation autophagosome maturation proceeds with recruitment of Microtubule‐associated protein light chain 3 (LC3) to PI3P‐containing early, pre‐autophagosomal membranes and modification of LC3 with a phosphatidylethanolamine (PE) lipid anchor (Dikic & Elazar, 2018). LC3 then drives cargo sequestration and autophagosome expansion. The Ulp family DUB‐like cysteine protease effector RavZ (Lpg1683) blocks this step (Choy et al., 2012; Horenkamp et al., 2015). RavZ localises to these membranes using a PI3P‐binding domain and sensing their unique curvature, interacts with LC3 via three eukaryotic‐like LC3‐interacting region (LIR) motifs, extracts the PE anchor from the membrane and cleaves it together with the C‐terminal glycine to which it is conjugated (Choy et al., 2012; Horenkamp et al., 2015; Kwon, Kim, Jung, et al., 2017; Yang, Pantoom, & Wu, 2017). Mammalian orthologues of LC3, such as GABARAPL1, which fulfil similar but non‐redundant functions, are also cleaved (Choy et al., 2012; Schaaf, Keulers, Vooijs, & Rouschop, 2016). The mechanism of action of RavZ was studied in great detail, showing that it requires RavZ‐membrane, RavZ‐PE lipid and RavZ‐LC3 interactions that are enhanced by a unique conformation of the LIR motifs generating higher binding affinity than classical LIR motifs and that are distinct from the binding mode of the cognate host protease ATG4, leading to irreversible rather than reversible delipidation (Kauffman et al., 2018; Kwon, Kim, Kim, et al., 2017; Park et al., 2021; Park et al., 2019; Yang, Pantoom, & Wu, 2020). While this an effective mechanism, a *L*. *pneumophila* Philadelphia *ΔravZ* strain still prevents sequestration of the LCV by autophagy, suggesting that this strain as well as *L*. *pneumophila* isolates, which do not encode RavZ, most likely deploy additional effectors to disrupt the process (Omotade, Roy, & Brodsky, 2020).

The host cytoskeleton is targeted by the *L*. *pneumophila* zinc‐dependent metalloprotease effector RavK (Lpg0969), which cleaves actin, rendering it unusable for polymerisation (Liu et al., 2017). The cleavage of actin is observed upon infection with *L*. *pneumophila* overexpressing RavK but not with wild‐type bacteria and the effector is dispensable for intracellular growth, leaving the role of its activity during infection uncertain.

Ectopic expression of the effector RavJ (Lpg0944) also perturbs the cytoskeleton, manifesting as the accumulation of F‐actin on the plasma membrane (Liu et al., 2017). Its overexpression is toxic for yeast and this can be controlled by the meta‐effector LegL1, which binds and seals the active site of RavJ (Urbanus et al., 2016). RavJ has two domains, an N‐terminal domain with papain protease fold and a C‐terminal domain that interacts with several septins in vitro (Urbanus et al., 2016). The catalytic residues of RavJ are essential for toxicity in yeast; however, Cys‐His‐Asp catalytic triads are also employed by other enzymes for different reactions, for example, the ubiquitin transfer by the ubiquitin E3 ligase effector SidC (Hsu et al., 2014), and actual protease activity and substrates of RavJ remain to be confirmed.

The crystal structure of the effector AnkH (LegA3) recently unveiled a cysteine protease‐like domain (Von Dwingelo et al., 2019), structurally resembling the *X*. *campestris* T3SS effector XpoD, a plant SUMO and ubiquitin isopeptidase (Peptidase C48, [Pruneda et al., 2016]). AnkH is conserved in all *Legionella* spp. and other bacteria that harbour a Dot/Icm T4SS, and a mutant is attenuated for intracellular growth (Gomez‐Valero et al., 2019; Habyarimana et al., 2008; Habyarimana, Price, Santic, Al‐Khodor, & Kwaik, 2010). AnkH interacts with the La‐related protein 7 (LARP7) component of the host cell transcription complex 7SK small nuclear ribonucleoprotein (snRNP), a complex involved in transcriptional elongation, impairing the interaction of LARP7 with this complex and triggering a global translational reprogramming that increases permissiveness of host cells to *Legionella* infection (Von Dwingelo et al., 2019). Full complementation of the attenuated *L*. *pneumophila* Δ*ankH* mutant requires the putative catalytic residues of AnkH, but LARP7 remains uncleaved and protease substrates unknown (Von Dwingelo et al., 2019).

## 6 EFFECTOR PROTEASES OF PHYTOPATHOGENS

Plants rely on two main cell autonomous innate immune mechanisms to recognise and fight invading pathogens, as they lack circulating immune cells and adaptive immunity (Jones & Dangl, 2006). Plant pattern recognition receptors (PRRs) are activated by PAMPs such as flagella, initiating PAMP‐triggered immunity (PTI). Pathogens disable PTI using effectors, but resistance (R) proteins guard this and activate ETI (Yuan, Ngou, Ding, & Xin, 2021). T3SS effector proteases are a major component of the virulence factor arsenal of phytopathogens and were reviewed recently (Figaj, Ambroziak, Przepiora, & Skorko‐Glonek, 2019; Mooney, Mantz, Graciet, & Huesgen, 2021). Nonetheless, due to their importance for illustrating the diversification of pan‐kingdom protease effector families and general concepts for activation and ETI, we highlight here some outstanding examples.

*Pseudomonas syringae* AvrPphB, a YopT‐like, peptidase C58 effector (Zhu, Shao, Innes, Dixon, & Xu, 2004), is produced as a pro‐protease that upon translocation undergoes autoproteolysis, allowing modification of unmasked myristoylation and palmitoylation sites with lipid moieties that tether the effector to membranes (Dowen, Engel, Shao, Ecker, & Dixon, 2009; Nimchuk et al., 2000; Puri et al., 1997; Shao et al., 2002). AvrPphB then cleaves the cytoplasmic kinase PBS1 and other PBS1‐like kinases including BIK1, PBL1 and PBL2 (Shao, Golstein, et al., 2003; Zhang et al., 2010), inhibiting PTI (Figaj et al., 2019). However, the R protein RPS5, which forms a complex with PBS1, detects its cleavage and induces ETI, leading to a hypersensitive response (HR) culminating in cell death and restriction of the pathogen.

Diverse phytopathogens encode AvrPphB homologues, for example, NopT, RipT, HopC1 and HopN1, some of which are subject to auto‐proteolytic cleavage and lipidation (NopT), just self‐cleave (RipT) or undergo neither modification (HopC1, HopN1) (Dai, Zeng, Xie, & Staehelin, 2008; Dowen et al., 2009). NopT from *Mesorhizobium amphore* targets ATP‐citrate synthase alpha chain protein 2 (ATP‐CSACP2) and hypersensitive‐induced response protein (HIRP) (Luo et al., 2020). HopN1 localises to chloroplasts and degrades PsbQ, a member of the oxygen‐evolving complex of photosystem II (PSII), inhibiting PSII activity as well as defence mechanisms such as the production of reactive oxygen species (ROS) (Rodriguez‐Herva et al., 2012). While substrates of other members still need to be identified, these peptidase C58 effectors highlight the striking diversification in function and activation mechanisms.

In addition, important plant protease effectors belong to the C70 and C103 peptidase families. AvrRpt2 (Peptidase C70) from *P*. *syringae* is activated by an unusual mechanism, requiring a host cyclophilin/ peptidyl‐prolyl isomerase, which licences auto‐proteolysis, releasing a stable product that is targeted to the plasma membrane (Axtell, Chisholm, Dahlbeck, & Staskawicz, 2003; Coaker, Falick, & Staskawicz, 2005; Coaker, Zhu, Ding, Van Doren, & Staskawicz, 2006; Mudgett & Staskawicz, 1999). AvrRpt2 acts on nitrate‐induced (NOI) domain‐containing proteins, in particular RPM1‐interacting protein 4 (RIN4), an important PTI signalling hub, and promotes auxin hormone signalling (Axtell et al., 2003; Bartho et al., 2019; Cui et al., 2013; Eschen‐Lippold et al., 2016; Goslin et al., 2019; Kim et al., 2005; Mackey, Belkhadir, Alonso, Ecker, & Dangl, 2003). Cleavage of RIN4 releases two fragments, which interfere with PTI, underlining the importance of cleavage products as signalling molecules. Given the central role of RIN4, it is not surprising that several ETI mechanisms detect the action of AvrRpt2 (Mooney et al., 2021).

Plant hormones beyond auxins, for example, salicylic acid (SA) and jasmonate (JA), are important PTI signals, and their modulation is a widely employed strategy of phytopathogens. Peptidase C103 effectors HopX1 (AvrPphE) and RipE1 degrade JAZ proteins, repressors of the JA pathway, resulting in suppression of SA‐induced defences, facilitating bacterial entry and replication; however, HopX1 is also counteracted by ETI in some plants (Gimenez‐Ibanez et al., 2014; Mooney et al., 2021; Nakano & Mukaihara, 2019; Z. L. Nimchuk, Fisher, Desveaux, Chang, & Dangl, 2007).

Phytopathogens, like animal pathogens, also harbour a large arsenal of CE clan proteases effectors, which are often DUBs removing ubiquitin and/or ubiquitin‐like modifiers, for example, XopD and AvrXv4. However, in particular, members of the peptidase C55 subfamily, for example, *Pseudomonas* HopZ1 and *Yersinia* effector YopJ, additionally or exclusively, can have acetyltransferase activity (Mooney et al., 2021; Pruneda et al., 2016). Notably, C55 peptidases XopJ of *X*. *campestris* and HopZ4 of *P*. *syringae* degrade an atypical substrate, ATPase 6 of the 19S regulatory particle of the 26S proteasome (RPT6), dampening SA‐mediated defences (Ustun, Bartetzko, & Bornke, 2013; Ustun & Bornke, 2015; Ustun, Konig, Guttman, & Bornke, 2014). While first structure‐function studies of bacterial CE proteases provided important insight (Pruneda et al., 2016), further investigation of the members of this effector family is required to fully understand the enzymology and substrate specificity of peptidase C55 effectors.

These examples illustrate that phytopathogens encode many protease effectors, which have often homologues in pathogens with different host ranges, and, thus, lessons learned about their enzymology and recognition by the host can be exemplary beyond plant‐pathogen interactions. It will be interesting to see if this promise holds true for *P*. *syringae* effector HopB1, which binds the plant immune receptor FLS2 to cleave the co‐receptor BAK1 and disrupt flagellin‐induced PTI using an unusual catalytic triad formed by a threonine, a histidine and two aspartates, making it the first threonine protease effector (L. Li et al., 2016).

## 7 CONCLUSION

Proteases are powerful enzymes, which irreversibly cleave proteins, and therefore control critical cell fate decisions. Bacterial pathogens integrated protease effectors in their virulence factor arsenals to harness this power. The effectors discussed here highlight their diversity and the effectiveness of this strategy. While they represent the best‐characterised examples, sequence‐based predictions suggest that dozens if not hundreds more await characterisation. Elucidation of their targets will shed new light on critical cellular processes that pathogens manipulate to exploit hosts. Moreover, while ETI is well‐established in plants, little is known about how protease effectors, depletion of their targets or newly generated fragments are perceived in mammalian cells. The intriguing discovery of NLRP1 as a sensor for anthrax lethal factor and viral protease activity (Chavarria‐Smith, Mitchell, Ho, Daugherty, & Vance, 2016; Tsu et al., 2021) supports the hypothesis that additional detection mechanisms exist and that future investigations of effector proteases will reveal exciting new concepts for the cellular microbiology and immunobiology of bacteria‐host interactions.

## CONFLICT OF INTEREST

The authors declare no potential conflict of interest.

## Data Availability

Not applicable ‐ no new data was generated for this literature review.
